# Imaging and quantification of human and viral circular RNAs

**DOI:** 10.1093/nar/gkae583

**Published:** 2024-07-25

**Authors:** Dabbu Kumar Jaijyan, Shaomin Yang, Santhamani Ramasamy, Alison Gu, Mulan Zeng, Selvakumar Subbian, Sanjay Tyagi, Hua Zhu

**Affiliations:** Department of Microbiology and Molecular Genetics, New Jersey Medical School, Rutgers University, 225 Warren Street, Newark, NJ 070101, USA; Department of Anatomy and Neurobiology, Virginia Commonwealth University, VA, USA; Guangdong Key Laboratory for Biomedical Measurements and Ultrasound Imaging, National-Regional Key Technology Engineering Laboratory for Medical Ultrasound, School of Biomedical Engineering, Shenzhen University Medical School, Shenzhen 518060, China; Department of Pain Medicine and Shenzhen Municipal Key Laboratory for Pain Medicine, Shenzhen Nanshan People's Hospital, The 6th Affiliated Hospital of Shenzhen University Health Science Center, Shenzhen, China; Public Health Research Institute, New Jersey Medical School, Rutgers University, 225 Warren Street, Newark. NJ 07103, USA; Department of Microbiology and Molecular Genetics, New Jersey Medical School, Rutgers University, 225 Warren Street, Newark, NJ 070101, USA; Department of Microbiology and Molecular Genetics, New Jersey Medical School, Rutgers University, 225 Warren Street, Newark, NJ 070101, USA; Public Health Research Institute, New Jersey Medical School, Rutgers University, 225 Warren Street, Newark. NJ 07103, USA; Public Health Research Institute, New Jersey Medical School, Rutgers University, 225 Warren Street, Newark. NJ 07103, USA; Department of Medicine, New Jersey Medical School, Rutgers University, USA; Department of Microbiology and Molecular Genetics, New Jersey Medical School, Rutgers University, 225 Warren Street, Newark, NJ 070101, USA

## Abstract

We present a robust approach for cellular detection, imaging, localization, and quantification of human and viral encoded circular RNAs (circRNA) using amplified fluorescence *in situ* hybridization (ampFISH). In this procedure, a pair of hairpin probes bind next to each other at contiguous stretches of sequence and then undergo a conformational reorganization which initiates a target-dependent hybridization chain reaction (HCR) resulting in deposition of an amplified fluorescent signal at the site. By harnessing the capabilities of both ampFISH and single-molecule FISH (smFISH), we selectively identified and imaged circular RNAs and their linear counterparts derived from the human genome, SARS-CoV-2 (an RNA virus), and human cytomegalovirus (HCMV, a DNA virus). Computational image processing facilitated accurate quantification of circular RNA molecules in individual cells. The specificity of ampFISH for circular RNA detection was confirmed through an *in situ* RNase R treatment that selectively degrades linear RNAs without impacting circular RNAs. The effectiveness of circular RNA detection was further validated by using ampFISH probes with mismatches and probe pairs that do not bind to the continuous sequence in their target RNAs but instead bind at segregated sites. An additional specificity test involved probes against the negative strands of the circular RNA sequence, absent in the cell. Importantly, our technique allows simultaneous detection of circular RNAs and their linear counterparts within the same cell with single molecule sensitivity, enabling explorations of circular RNA biogenesis, subcellular localization, and functions.

## Graphical Abstract

**Graphical Abstract:**
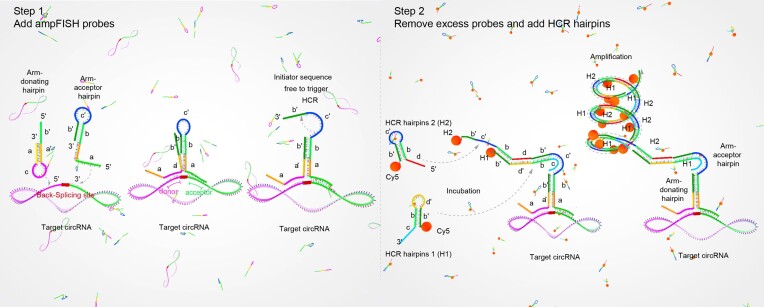

## Introduction

Circular RNAs are a unique form of RNA that contains covalently closed ends (1). The 5′ cap and the 3′ tail are absent in circular RNA, making them resistant to exonucleases like RNase R (2). Circular RNAs have been associated with many diseases including diabetes, neurodegenerative diseases, osteoarthritis, cancer, aging-associated diseases, cardiovascular disease (3–10). Studies have also found that circular RNAs can be used as biomarkers for many diseases including cancers, neurological diseases, and autoimmune diseases (10–12).

The biogenesis of circular RNAs is a highly regulated process, which is controlled by trans-(splicing factors) (13–15) or *cis-*(intronic complementary sequences) regulatory elements that help in back-splicing (16–18). It has been reported that circular RNAs are specific to cells and tissues and are mostly conserved in mammalian cells. Some circular RNAs are present in larger amounts than their respective linear forms (19,20). Despite the advances in RNA biology, we have limited knowledge about the functions and biogenesis of circular RNAs. One of the most notable functions of circular RNAs is their ability to effectively sponge microRNAs (12). Another function of circular RNAs is binding to regulatory proteins, which is often seen in the context of cancer (21,22). Not only do circular RNAs play a vital role in various diseases like cancer but they also have a crucial role in viral pathogenesis (23,24).

Next-generation sequencing is currently the mainstay for identification of circular RNAs (25,26). A better understanding of the functions and mechanism of biogenesis of circular RNAs will result from techniques that provide information about their intracellular distributions. Subcellular fractionation of cytoplasmic and nuclear components followed by qPCR or RNAseq are often used to study circular RNA localization. However, getting pure cytoplasmic and nuclear fractions is difficult to obtain and it is desirable to have better resolution. Conventional fluorescence *in situ* hybridization using biotin labeled RNA probes followed by tyramide signal amplification have successfully been used for detection of a circular RNA (25). In addition, two studies have utilized single molecule fluorescence in situ hybridization (smFISH) to detect circular RNAs (27,28). In smFISH about 50 small oligonucleotides labeled with a single dye are bound to the same RNA. Deposition of so many dye moieties on the same mRNA molecule renders each molecule so intensely fluorescent that it becomes visible as a diffraction-limited spot under a fluorescence microscope (29). Kocks et al 2018 used smFISH to image CDR1 RNA, which is expressed only as a circular isoform and has no linear counterpart. However, this approach is useful only in few cases where there is no linear isoform of a circular RNA with overlapping sequence. This limitation was overcome by Kopulla et al 2022, who used two color probes sets where one probe set is targeted to a sequence that is common to circular RNA and its linear counterpart and the second probe set labeled, in a second color, is targeted to a sequence present only in the linear RNA. In this strategy, linear RNA is visible in both colors whereas circular RNA is visible in just the first color. Although image analysis permits specific detection of circular RNA, the circular topology is not specifically detected in this strategy. Another limitation of this approach is that when the circular RNA is less than about 500 hundred nucleotides, sufficient number of smFISH probes can’t be designed to obtain detectable signals.

A second strategy does focus on the circular topology of circular RNA by detecting sequences that come together at the back-splice junction (BSJ). This technique utilizes RNAscope technology (30) where two ‘Z’ probes must bind at adjacent locations on the same RNA in order to yield an amplified branch DNA signal (31). This strategy is effective, and yields amplified signal, but how close the target binding regions of pair of Z probes must be to each other to yield a signal is not known. Only requirement in the double Z strategy is that the two Z probes should bind to the same target and the label so that the complex has higher stability than possible with individual Z probes. The design algorithm for RNAscope Z probes is proprietary, they are only available commercially and are expensive.

Here, we demonstrate an alternative in situ circular RNA detection approach that is based on high fidelity ampFISH (32). In ampFISH, a pair of interacting hairpin probes are utilized for the detection of stretches of sequences that are immediately adjacent. When this pair of probes bind at these adjacent sites, they interact via a strand displacement reaction and unmask a sequence that is sequestered in the free probes, which creates an initiator for hybridization chain reaction (HCR) (33–35). HCR creates a polymer of labeled hairpins and deposits it at the site where the pair of hairpins are bound, creating a spot-like signal that can be visualized in a fluorescence microscope. In contrast with the Z-probe system, the ampFISH requires that the two probes must be immediately adjacent to yield a signal (see Results). Specificity and amplifications characteristics of ampFISH have been described earlier (32).

Through bioinformatic and experiential approaches we have found that coronaviruses, SARS-CoV-2, encode hundreds of circular RNAs (11). The circular RNAs encoded by coronavirus were more abundant and longer compared to circular RNAs encoded by the host-genome (11). These RNAs are likely to be created by RNA structure-mediated template switching during the replication process (11,36). In addition to the coronaviruses, our sequencing analysis of human cytomegalovirus (HCMV) infected cells revealed that HCMV genome also encodes many circular RNAs (26).

In this study, we employed ampFISH to detect circular RNAs and smFISH for the detection of their linear counterparts. We show in situ detection of a well-characterized human circular RNA, a SARS-CoV-2 encoded circular RNA, and an HCMV encoded circular RNA. We demonstrated the specificity of detection by treating the cells with RNase R in situ prior to hybridization and by employing a number of different negative control probes sets. Single-molecule multiplex imaging of both circular and linear RNAs was accomplished using distinguishable labels. Although some of the results shown here have previously been presented elsewhere (26,36), this report provides the full evidence of specificity of detection, provides experimental details, and a comparison with other closely related technologies. These results will aid in future studies aimed at the exploration of circular RNA biogenesis and function.

## Materials and methods

### Cells and viruses.

MRC-5 cells (ATCC, CCL-171), Vero-E6 cells (ATCC), and Human retinal pigment epithelial cells (ATCC, ARPE-19-CRL-2302) were grown in Dulbecco's Modified Eagle Medium (DMEM, Gibco) supplemented with 10% fetal bovine serum (FBS, HyClone) and 1% penicillin & streptomycin (P&S). MRC-5 cells were used to culture Towne-GFP (HCMV). The Vero-E6 cells were used to culture SARS-CoV-2 (Wuhan strain) in the BSL-3 facility at Public Health Research Institute (PHRI), Rutgers University, New Jersey. Virus infections were performed in DMEM with 5% FBS and 1% P&S.

Viral infections and cell preparations. Cells were seeded on the glass coverslips (0.17 mm thick). Before seeding the cells, the coverslips were coated with 0.1% gelatin (Sigma-Aldrich, cat. no. G939-100G) for 20 minutes at 37 ᵒC followed by 3 washes with 1× PBS (prepared from 10x PBS pH 7.4, Gibco, cat. no. 70011-044). MRC-5 cells were infected with Towne-GFP virus at a multiplicity of infection (MOI) of 0.05 and coverslips were removed at 48 hours post-infection and fixed. For fixation, we used 4% paraformaldehyde (prepared from 32% Paraformaldehyde solution obtained from Fisher Scientific cat. No. 50–980-495) in 1× PBS. Subsequently, they were permeabilized with 70% ethanol (Fisher Scientific, cat. no. S25309B) for 10 minutes at room temperature and either used immediately or stored at –20°C. SARS-CoV-2 infection was carried out at MOI of 0.1 in Vero cells seeded on glass coverslips. The infected cells were fixed 24 hours post-infection, permeabilized in 70% alcohol as above, and then transported outside the BSL3 facility.

### RNase treatments

We used 3′ exonuclease, RNase R (Lucigen, LGC Biosearch Technologies, Petaluma, CA), to selectively degrade the linear RNAs *in situ* while preserving the circular RNAs (37). For RNase R treatment, the cells were first washed with 1× PBS, treated with 1× PBS supplemented with 0.5% Triton X-100 for 10 minutes at room temperature, and equilibrated with 1× RNase R buffer for 30 minutes at 37°C. The RNase R treatment was given for 4 hours at 37 °C using 10 units of RNase R per coverslip in a 50 ml reaction. In the case of SARS-CoV-2 infected Vero cells this incubation was extended to 8 hours because due to high abundance of SARS-CoV-2 RNA signals from linear RNAs persisted in the short incubations.

We performed RNase A treatment to degrade both linear and circular RNAs for one of the controls. Cells were washed with 1× PBS, treated with 1× PBS supplemented with 0.5% Triton X-100 for 10 min at room temperature, and equilibrated with 1× RNase R buffer for 30 min at 37°C. The RNase A treatment was performed in 1× RNase R buffer for 2 h at 37 °C using 10 μg per coverslip in 50 ml reaction.

### ampFISH and smFISH probes

The probes were designed to target sequences that are contiguous at the back-spliced junction in the circular RNA, but are located at distant regions in the linear RNA counterpart. The donor and the acceptor probes were designed to meet at the back-splicing junction. The lengths of each probe segment were chosen to ensure the stability of the probe target hybrid under the hybridization conditions. Hairpin elements, interacting with each other upon binding to the target, were incorporated into the probe sequences, as illustrated in Table 1. All ampFISH probe pairs were designed to generate signals through HCR hairpins 1 to 4 as described earlier by Marras et al. 2019 (32).

**Table 1.** Probe sequences and HCR hairpins used in this study. The hairpin stems are underlined and target complementary sequences are bolded. Abbreviations are: DP, donor probe; AP, acceptor probe

Probe Name	Organism	Sequence
DP-positive	HCMV	GTTACAGACGACTCCCACAGTCC**CGAGGGGATATAAATCACCG**GACT
AP-positive	HCMV	**GAGGGCTATGTTTTTTGCTATGTACG**GGACTGTGGGAGTCGTCTGTAACTACTTCATGTTACAGACGACTCCCAC
DP-negative	HCMV	GTTACAGACGACTCCCACAGTCC**CGTACATAGCAAAAAACATA**GGACT
AP-Negative	HCMV	**CTCGACGGTGATTTATATCCCCTCG**GGACTGTGGGAGTCGTCTGTAACTACTTCATGTTACAGACGACTCCCAC
DP Linear 29489	HCMV	GTTACAGACGACTCCCACAGTCC**CGAGGACACCGCCGTCTACT**GGACT
AP-Linear-29489	HCMV	**GAGGGCTATGTTTTTTGCTATGTACG**GGACTGTGGGAGTCGTCTGTAACTACTTCATGTTACAGACGACTCCCAC
DP circHIPK3(2)-ve	Human	GTTACAGACGACTCCCACAGTCC**GTATGGCCTCACAAGTCTTGGTCT**GGACT
AP circHIPK3(2)-ve	Human	**ATATCTACAATCTCGGTACTACAG**GGACTGTGGGAGTCGTCTGTAACTACTTCATGTTACAGACGACTCCCAC
DP circHIPK3(2)+ve	Human	GTTACAGACGACTCCCACAGTCC**CTGTAGTACCGAGATTGTAGATAT**GGACT
AP circHIPK3(2)+ve	Human	**TAGACCAAGACTTGTGAGGCCATAC**GGACTGTGGGAGTCGTCTGTAACTACTTCATGTTACAGACGACTCCCAC
DP linear HIPK3 Exon-3–4	Human	TAGGT**AGTCTGAGAAATGTATCGAAT**ACCTACCTCGTAAATCCTCATCAATCATC
AP linear HIPK3 Exon-3–4	Human	CCTCGTAAATCCTCATCAATCATCCAGTAAACCGCCGATGATTGATGAGGATTTAGGAGGTAGGT**CTGATCATACTCCAAGGCTCCTG**
DP COVID (-)	SARS-CoV-2	GTTACAGACGACTCCCACAGTCC**AATCAGCGAAATGCACCCCGCATT**GGACT
AP COVID (-)	SARS-CoV-2	**AGACGTGGTCCAGAACAAACCCAA**GGACTGTGGGAGTCGTCTGTAACTACTTCATGTTACAGACGACTCCCAC
DP COVID (+)	SARS-CoV-2	GTTACAGACGACTCCCACAGTCC**TTGGGTTTGTTCTGGACCACGTCT**GGACT
AP COVID (+)	SARS-CoV-2	**AATGCGGGGTGCATTTCGCTGATT**GGACTGTGGGAGTCGTCTGTAACTACTTCATGTTACAGACGACTCCCAC
DP COVID (+) control	SARS-CoV-2	GTTACAGACGACTCCCACAGTCC**TTGGATTTGGTCTGCACCATGTCT**GGACT
AP COVID (+) control	SARS-CoV-2	**AATACGGGCTGCAATTCCCTGGTT**GGACTGTGGGAGTCGTCTGTAACTACTTCATGTTACAGACGACTCCCAC
DP COVID (+) -control	SARS-CoV-2	GTTACAGACGACTCCCACAGTCC**CGAAAGCTTGTGTTACATTGTATG**GGACT
HCR hairpin H1		GGCGGTTTACTGGATGATTGATGAGGATTTACGAGGAGCTCAGTCCATCCTCGTAAATCCTCATCAATCATC-TMR
HCR hairpin H2		TMR-CCTCGTAAATCCTCATCAATCATCCAGTAAACCGCCGATGATTGATGAGGATTTACGAGGATGGACTGAGCT
HCR hairpin H3		Cy5-ACAGACGACTCCCACATTCTCCAGGTGGGAGTCGTCTGTAACATGAAGTA
HCR hairpin H4		CTGGAGAATGTGGGAGTCGTCTGTTACTTCATGTTACAGACGACTCCCAC-Cy5

The donor and acceptor probes were sourced from Sigma-Aldrich, then purified through gel electrophoresis employing an 8% polyacrylamide gel (prepared using 30% acrylamide/bis solution, BIO-RAD, cat. no. 1 610 154) with 8M urea (Sigma-Aldrich, cat. no. U5378). The gel section containing the DNA was excised, and DNA was eluted using a gel elution buffer (400 mM NaCl, 20 mM Tris pH 8.0, and 1 mM EDTA) by shaking overnight at room temperature. Subsequently, the gel-purified probes underwent alcohol precipitation and were stored. The ampFISH probes were diluted in 2× SSC buffer (prepared from 20× SSC Buffer, ThermoFisher, cat. no. 15557-044 and RNase-free water, ThermoFisher cat. no. 10977-015) at a concentration of 2.5 μM and snap-cooled by heating at 100°C, followed by returning to room temperature for 5 min.

To assess the specificity of ampFISH probes, we employed a pair of acceptor and donor probes that were designed to specifically recognize linear RNA while excluding circular RNA. These probe pairs were crafted based on the exon-intron boundaries that are adjacent in the linear RNA but absent in circular RNA. A pair of probes targeting the negative strand of circular RNA was also designed for the detection of negative strand circular RNA. Additional control probes, comprising acceptor and donor probes with three random mutations, were also designed. Furthermore, we developed another pair of acceptor and donor probes binding 20–50 nucleotides away from the back-splice junction region of circular RNA. For virus-encoded circular RNA, virus-infected cells served as a positive control, while uninfected cells served as a negative control. In both ampFISH and smFISH, RNA spots typically exhibit brightness, enabling easy discrimination from diffuse backgrounds or cell debris.

For smFISH, 48 oligonucleotide probes were designed for HIPK3 RNA and for the ORF1a of SARS-CoV-2, using the Stellaris probe designer program available at http://www.singlemoleculefish.com. Sequences of smFISH probes for HIPK3 RNA and SARS-CoV-2 ORF1a are provided in Supplementary Table S1. We obtained oligonucleotides with 3′-amino modification from LGC Biosearch Technology (Petaluma, CA), pooled them in equimolar amounts, conjugated them with amino reactive dyes (Texas red), and then purified the coupled oligonucleotides as described earlier (29,32).

### Hybridization, HCR and Imaging

smFISH was performed either alone or in combination with ampFISH. Coverslips with cells were withdrawn from 70% alcohol and equilibrated with 1 ml of hybridization wash buffer (2× SSC, 10% formamide (Ambion, cat. no. AM9342)) for 10 min at room temperature before hybridization. Hybridization reaction mixtures were typically 50 μl and contained: 10% dextran sulfate (Sigma-Aldrich cat. no. D8906), 1 mg/ml tRNA from baker's yeast (Sigma-Aldrich, cat. no. R8759), 2 mM ribonucleoside vanadyl complexes (New England Biolabs, Ipswich, MA), 0.02% ribonuclease-free bovine serum albumin (ThermoFisher Scientific), 10% formamide, 2× SSC, and probes that varied from experiment to experiment. When performing co-imaging with smFISH and ampFISH probes, we added 1 μl of smFISH probes (25 ng/μl) and 2 μl each of donor and acceptor probes (10 ng/μl) to the 50 μl hybridization mixture. When ampFISH was performed alone, the smFISH probes were omitted. This mixture was placed on a parafilm stretched over a glass sheet placed in a tip box that contained water in its bottom. The coverslips were placed over the droplet with cells facing down and incubated in a water bath at 37°C overnight. Coverslips were floated by introducing 100 μl of hybridization wash buffer and then transferred to 12 well plates with cell sides facing up. Excess probes were removed by three washes with hybridization wash buffer (1 ml each). Subsequently, HCR was performed in HCR buffer (50 μl) that consisted of 5× SSC, 10% dextran sulfate, 0.05% Tween 20, and 125 nM of each HCR hairpin at 25 ºC for 2–4 h. To remove excess HCR hairpins, the coverslips were washed twice with the hybridization wash buffer followed by a wash with a glucose buffer (2× SSC, 0.4% glucose). The coverslips were mounted in 7–10 μl of a deoxygenated medium (to minimize photobleaching), which was prepared immediately before use by adding 1 μl of 3.7 mg/ml glucose oxidase and 1 μl of catalase suspension (both from Sigma-Aldrich, Cat. nos. G6125 and C3515-25MG), 1 μl of DAPI into 100 μl glucose buffer. After withdrawing the excess of mounting medium by blotting with Kimwipes, the edges of coverslips were sealed with clear nail polish and imaged the same day.

We usually performed ampFISH using one-step procedure where both donor and acceptor probes were used together. To ensure that the signals are specific, we explored a two-step ampFISH procedure where first acceptor probe was hybridized and after removal of the excess acceptor probes, the donor probes are hybridized. The two step procedure yielded equivalent results.

The images were acquired using an Axiovert 200M inverted fluorescence microscope (Zeiss, Oberkochen, Germany) using a 63× oil immersion objective (numerical aperture 1.4) and a Prime sCMOS camera (Photometrics, Tucson, AZ) controlled by MetaMorph image acquisition software (Molecular Devices, San Jose, CA). We acquired images in 16–20 optical z-sections separated from each other by 0.2 μm at 100- to 3000-millisecond exposure times in each fluorescence color channel. Z-sectioning is important for capturing all mRNAs as some spots lie outside single focal plains.

### Co-imaging of circular and linear RNAs

We hybridized both kinds of probes (smFISH and ampFISH) together in a common overnight incubation. The next day excess primary probes were removed by washing and the coverslips were subjected to HCR. HCR yielded signals in a different color (Cy5) than the smFISH (Texas Red). The smFISH probes remain stably bound during the HCR amplification and wash steps (which are carried out at room temperature and under permissive hybridization conditions). Finally, the signals from the two kinds of probes are imaged together.

For mRNA spot recognition and counting we used a custom image processing program written in MATLAB (MathWorks, Natick, MA) environment. First introduced by Raj *et al.* (29), this program has been used successfully in many studies for RNA spot analysis, and current versions are deposited at https://github.com/TyagiLab/CountRNASpots. In these algorithms, we first combine a DAPI and a bright field image and then use the combined image to manually draw cell boundaries. Thereafter, we select thresholds in the fluorescence z-stack images for segmentation of RNA spots in 3D. In each image, the results of spot identification are manually confirmed for accuracy.

### Statistics and figure preparation

Data is represented as a mean and the error bars represent standard error. Two-tailed unpaired *t*-test was used to compare the RNA spots in infected and uninfected cells. Images were prepared in ImageJ, sized in Photoshop, and then transferred to Illustrator, where they were integrated with vector graphics drawings.

## Results

### Detection, localization, and quantification of a human circular RNA in situ

**Figure 1.**
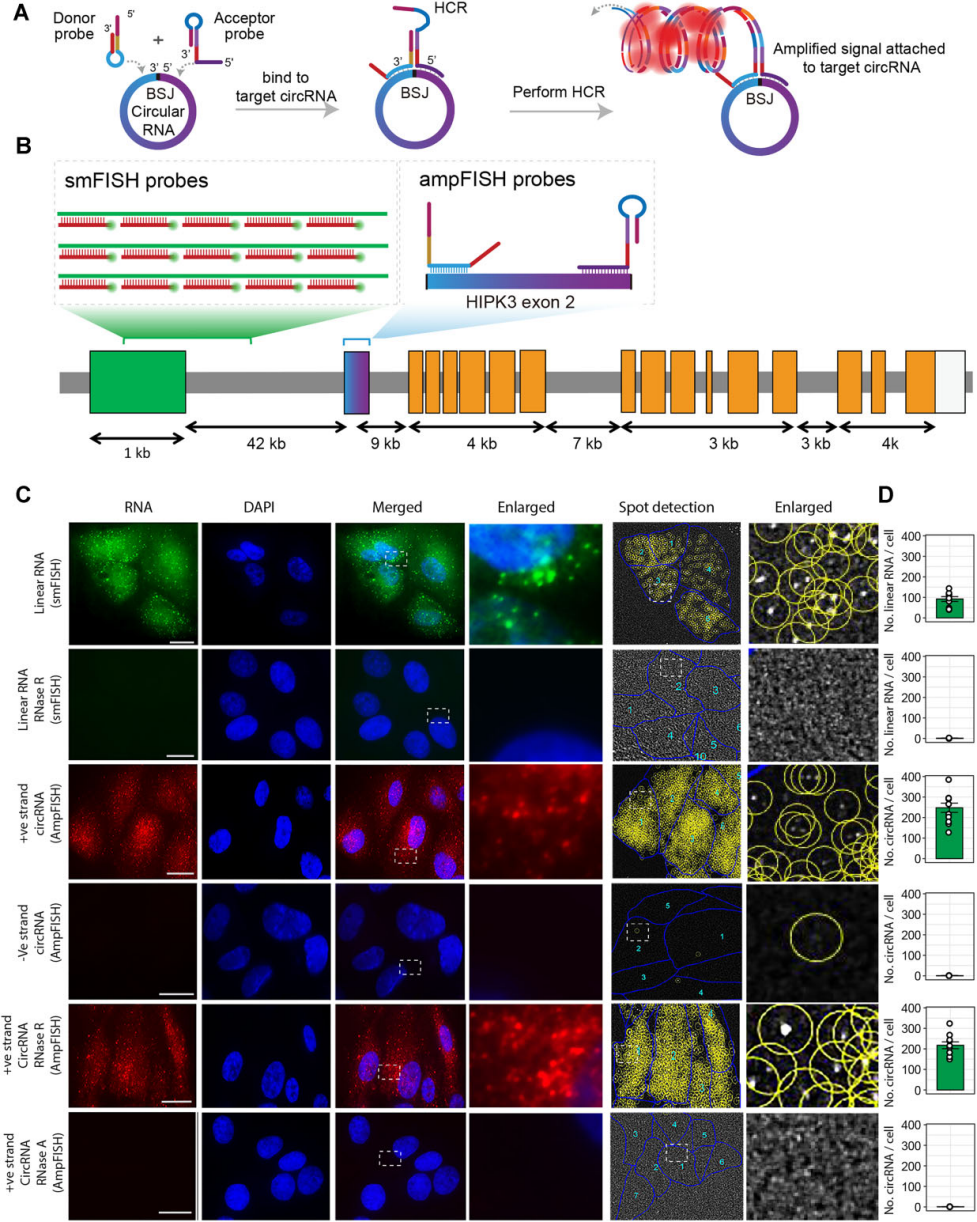
Imaging circular and linear RNAs encoded by human gene HIPK3 using ampFISH and smFISH. (**A**) A Schematic description of how ampFISH is used to detect contiguous sequences at back-splice junction (BSJ) in the circular RNAs. When bound to the circular RNA at BSJ the ampFISH probe pairs are juxtaposed and are able to interact with each other, causing a conformational reorganization which releases an HCR initiator sequence. The freed initiator creates amplified signals when HCR hairpins are provided. (**B**) Organization of human HIPK3 gene and the locations of the smFISH and ampFISH probes. The ampFISH probe pairs can bind to the terminal sequences on exon 2 in the linear RNA but cannot interact with each other and therefore do not generate any HCR signals from linear RNA. smFISH probes are directly labeled and 48 probes are tiled over exon 1. (**C**) Imaging of linear and circular HIPK3 RNAs with smFISH and ampFISH. The probes, the targeted RNA species, and the conditions are indicated on the left of each row of images, and the image components are shown on the top. DAPI represents the nucleus of the cells. The white dotted lines represent the enlarged areas. Two columns of images on the right show spot detection analysis where yellow circles are drawn around each detected spot and the blue lines represent cell boundaries. Each spot corresponds to single RNA molecule. smFISH signals are shown by green and ampFISH signals are shown by red color. The bottom panel represents the RNase A treatment of cells followed by ampFISH staining to detect positive strand circRNA of HIPK3. (**D**) The bar graphs show quantification of number of spots per cell from several fields of images. The size bars represent 20 μm. Data represent mean RNA copies/cell ± SE. At least 10 cells were analyzed in each category.

A schematic diagram describing ampFISH for circular RNA detection in situ is shown in Figure 1A. In amplified FISH (ampFISH) two interactive hairpin shaped oligonucleotide probes (donor and acceptor probes) that bind to contiguous sequences in the target are used. The simultaneous binding of the two probes next to each other initiates a strand displacement interaction between them that results in a conformational reorganization in the acceptor probe which renders a previously double-stranded region within it single-stranded (32). The target mediated revelation of this sequence allows initiation of a hybridization chain reaction (HCR) which creates a labeled multimer of the HCR hairpins at the target's location which can be detected. The probes that bind to nonspecific sites don’t initiate this reaction.

Since sequences that are distal in the linear RNAs become contiguous in circular RNAs, ampFISH is well-suited for detection of circular RNAs. To demonstrate the efficacy of ampFISH for circular RNA detection, we first used the example of a circular RNA derived from the HIPK3 gene, circHIPK3(2) (the number within the bracket indicates the number of exon that is present in the circular RNA following a recent nomenclature system) (38). This RNA is well expressed and has been characterized before (25,39). The sequence that encodes circHIPK3(2) is located on chromosome 11 at nucleotide position 33307958–33309057 on the positive strand (circBank ID: hsa_circHIPK3_004). circHIPK3(2) and is generated from exon 2 of the HIPK3 gene. circHIPK3(2) has been detected in many types of cells (25) and has been implicated to play a role in a number of cellular processes including cellular homeostasis, cell proliferation, apoptosis, miRNA sequesteration, and cell cycle (39–43).

We designed a pair of ampFISH probes for the sequences present at either side of BSJ in circHIPK3(2). In addition, as a control, we prepared a pair of ampFISH probes that target the complement of circHIPK3(2) (-ve strand probes). The sequences of ampFISH probes are presented in Table 1 where target complementary regions are indicated by bold residues. We also designed a set of 48 smFISH probes for the linear counterpart of circHIPK3(2) while avoiding selection of probes that would bind to exon that corresponds to circHIPK3(2) (locations of smFISH probes shown in Figure 1B and their sequences are presented in Supplementary Table S1). The linear RNA-specific smFISH probes were labeled with Texas Red and the ampFISH probes elicit signals from HCR hairpins labeled with Cy5 so that they can be distinguished from each other.

These probes were separately hybridized to Human retinal pigment epithelial cells, ARPE-19. In addition to hybridizing to the fixed and permeabilized ARPE-19 cells under normal conditions, we pretreated these cells (on separate coverslips) with either RNase R or RNase A, the former is a 3′-5′ exonuclease which digests linear RNAs but spares the circular RNAs and the latter is an endonuclease and digests all RNAs. These *in situ* digestions served as important negative controls. After hybridization, excess probes were removed, and in the case of coverslips which contained ampFISH probes, HCR amplification was carried out. After several hours of HCR, the excess HCR hairpins were removed and all coverslips were imaged while acquiring z-sections through the volume of the cells.

Representative compressed z-stacks are presented in Figure 1C. The results show that both linear RNA-specific smFISH probes and circular RNA-specific ampFISH probes yield spot-like signals in coverslips under normal conditions (Figure 1 C). However, when RNase R was used against the linear RNAs, the signal from linear mRNA-specific smFISH was lost. The ampFISH probes against the negative strands of circHIPK3(2) did not yield any signals. Finally, there was no signal from circHIPK3(2) when RNase A was employed. The spots in each image were detected in the full volumes of the cells using an in-house image processing algorithm (Figure 1D). The average counts of spots (each spot corresponds to a single RNA molecule) in single cells are presented in bar graphs in the right in Figure 1. These results show that the number of circHIPK3(2) RNA molecules is more than its linear counterpart which has been observed in previous PCR studies with outward facing primers (25), and that the signals for circHIPK3(2) RNA are specific in the light of the controls. These experiments for circHIPK3(2) detection were repeated in MRC5 cells, and similar results were obtained (Supplementary Figure S1). Enlarged merged images of each panel as provided in Supplementary Figure S2 were used to visualize circular RNA spots in the stained cells.

### Co-imaging of circular RNA and linear RNA

**Figure 2.**
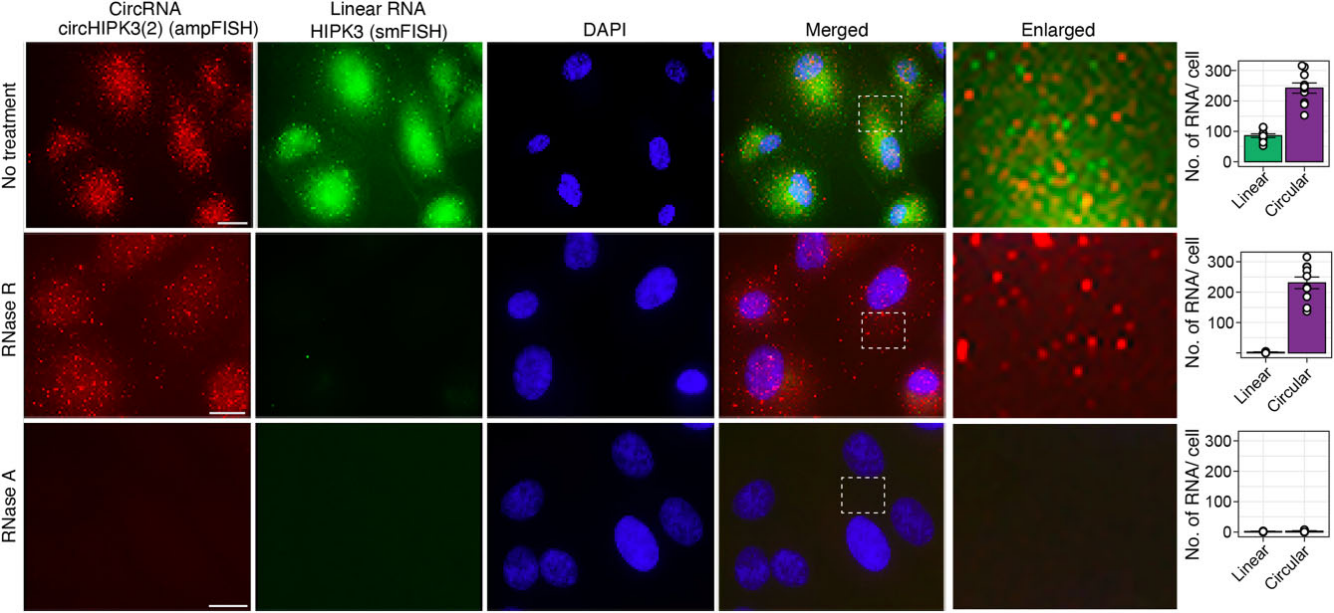
Co-imaging HIPK3 circular and linear RNAs. The circular RNA was imaged using ampFISH probes (red signals) and the linear RNA was imaged using smFISH probes (green signals). The probe locations are depicted in Figure 1. Both kinds of probes were hybridized together and after removal of excess probes by washing, HCR was performed. The cell conditions are indicated on the left and the detected RNA species on the top. DAPI was used to stain nuclei. The enlarged images are the magnified from the selected area of the merged image (white dotted line). Number of spots detected in single cells in each category are shown on the right. The scale bars represent 20 μm. Data are mean number of RNAs per cell ± SE, *n* = 5 cells.

In order to demonstrate the selectivity of RNase R in removing signals from linear RNA while preserving the signals from the circular RNA, we performed smFISH and ampFISH simultaneously on the same cells. The results presented in Figure 2 indicate that RNase R treatment abolishes the linear RNA spots but the circular RNA spots remained intact. As expected, the RNase A treatment abolished signals from both kinds of RNAs. The images also showed that the spots from linear RNA do not colocalize with the spots from circular RNAs, which further confirms the specificity of signals. To support these conclusions, we present some enlarged images of linear and circular RNAs in Supplementary Figure S3. We observed that the circHIPK3(2) was present in both cytoplasm and nucleus of ARPE-19 cells with majority being localized in the cytoplasm, in contrast, the linear RNA was localized in cytoplasm (Figures 1C and 2).

In order to demonstrate the detection of both circular and linear RNAs with ampFISH in the same cells, we designed a pair of ampFISH probes for the exon-exon junction between HIPK3 exons 3 and 4. The sequences of probes are provided in Table 1. To confirm the identities of the ampFISH signals from the linear RNA in this experiment, we relied upon their expected colocalization with the smFISH signals from 48 probes bound to the rest of the linear RNA transcript. We therefore performed triplex imaging where the tetramethyl rhodamine (TMR) channel was dedicated for exon 3–4 ampFISH, the Texas Red channel for linear RNA smFISH, and the Cy5 channel for circHIPK3(2) ampFISH. These experiments were performed in HeLa cells.

**Figure 3.**
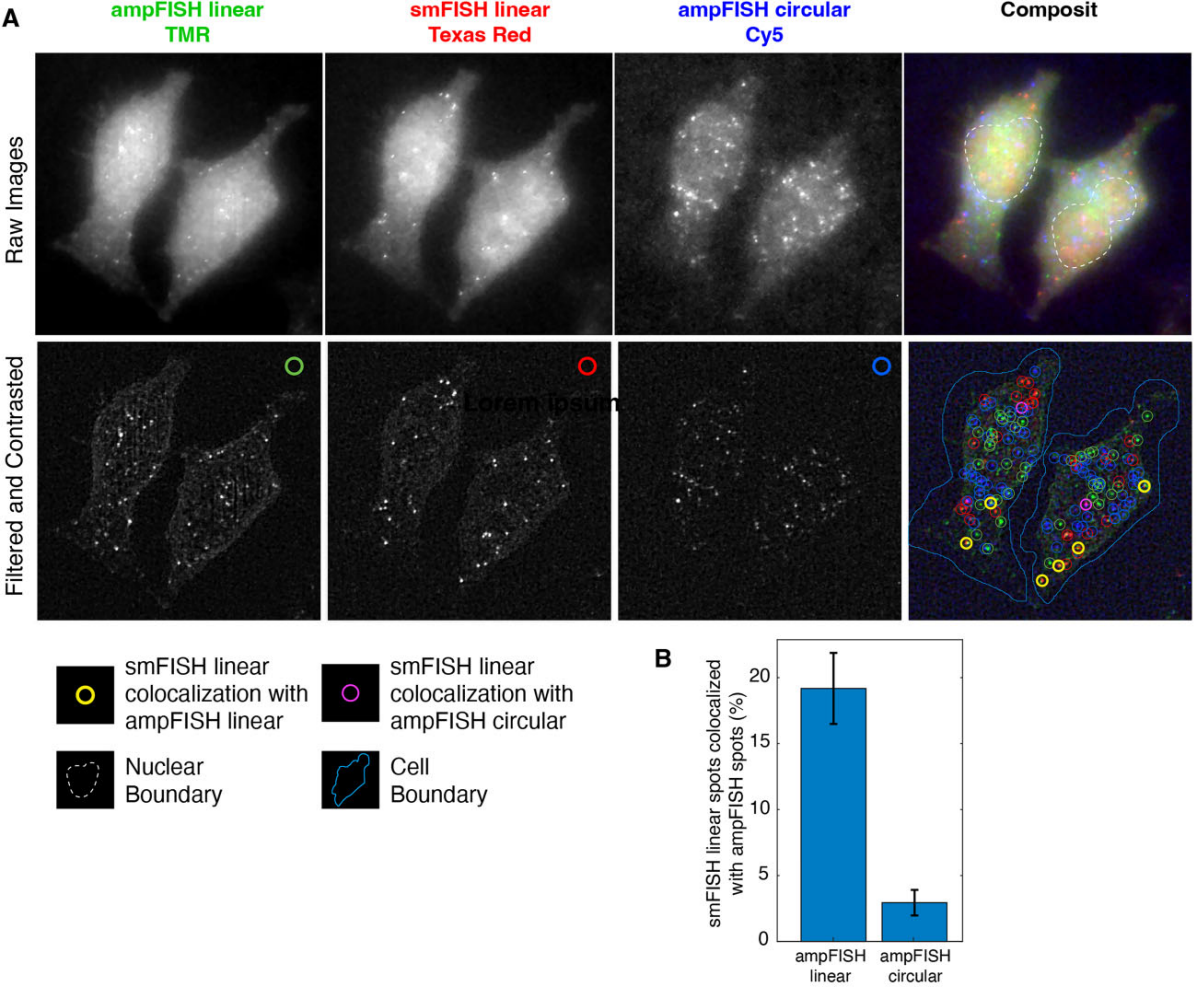
Detection of HIPK3 linear transcripts using a pair of ampFISH probes targeted at the junction between exons 3 and 4 and the HIPK3 circular transcripts using a pair of ampFISH probes targeted at their back-splice junction (BSJ). The former is confirmed by using 48 smFISH probes against the linear transcripts in the same images. (**A**) Results of a triplex imaging experiment are shown where top panels correspond to the unprocessed z-stack merges from the indicated channels and the bottom panels are obtained by application of a Laplacian filter on the z-stack layers and merger of the layers followed by contrasting. Right most panels are composite images of the three channels created using color codes indicated by the labels above or over the images. In the bottom right panel, circles of different colors identify the spots that were detected by our image processing algorithm in each channel. The yellow and purple colored circles identify the smFISH linear spots that were localized with either ampFISH linear and ampFISH circular, respectively, the latter can arise only spuriously. (**B**) Percent of smFISH linear spots that were colocalized with spots created by the ampFISH linear or ampFISH circular probes. Number of cells analyzed 68. Error bars represent 95% confidence interval.

We observed that on average there were 27.5 (± 2.8 CI) TMR spots, 16.9 (± 1.6 CI) Texas Red spots, and 17.6 (± 2.4 CI) Cy5 spots in single cells (Figure 3). Pairwise co-localization analysis between TMR and TR spots and Cy5 and TR spots revealed that while 19.1% of TR spots were localized with TMR spots, only 2.7% of TR spots were colocalized with Cy5 spots (Figure 3 B). Our analysis procedure that has been described earlier (44), classifies spots in different channels as colocalized when they emanate from the same RNA molecule. Since the efficiency of detection by smFISH is high (about 85% of mRNAs can be detected) (29), this indicates that about 19.1% of HIPK3 linear transcripts can be accurately detected by ampFISH probes. This efficiency is consistent with earlier results where single ampFISH and multiple smFISH probes were used to detect the same mRNA (32). In contrast, the circular RNA spots (Cy5) were generally not colocalized with the linear mRNA spots (TR). However, we observed that a fraction of TMR spots were extra, i.e. not co-localized with the TR spots, which might correspond to noise or to other HIPK3 isoforms not detected by our smFISH probe set.

The finding that linear HIPK3 RNA is present in similar numbers as circular HIPK3 RNA (16.9 spots versus 17.6 spots per cell) in HeLa cells is seemingly at odds with previous observations which suggest that circHIPK3(2) is present at higher levels than its linear counterpart (25). However, as discussed above, ampFISH detects only about 20% of RNAs that are present in the cell, which will indicate about five times as many circular RNAs as the linear RNAs. When we extend the similar reasoning to ARPE-19 cells (Figure 1), the circular RNA would be about 12-times as much as the linear RNA.

Overall, these results demonstrate that ampFISH probes can be used to detect contiguous pair of target sequences *in situ* such as in the case of circular RNA BSJ.

### Imaging a SARS-CoV-2 derived circular RNA in infected cells.

Through bioinformatic analysis of transcriptome of SARS-COV-2 infected cells, we have recently shown that many circular RNAs are produced by this virus (36). Other coronaviruses like SARS-CoV and MERS-CoV also produce many circular RNAs (36). We confirmed the presence of several circular RNA species in SARS-COV-2 through their resistance to RNase R followed by PCR amplification across BSJ. These circular RNAs are not produced during splicing, since the virus spends its entire life cycle in the cytoplasm and does not experience normal nuclear splicing, instead, they are likely to be produced by homology mediated template switching events during the viral replication (36). We sought to detect one of the well-characterized SARS-CoV-2 circular RNAs, NC_045512.2:29 122|28 295, using ampFISH. This RNA is encoded in the N-gene region of the SARS-CoV-2 genome.

**Figure 4.**
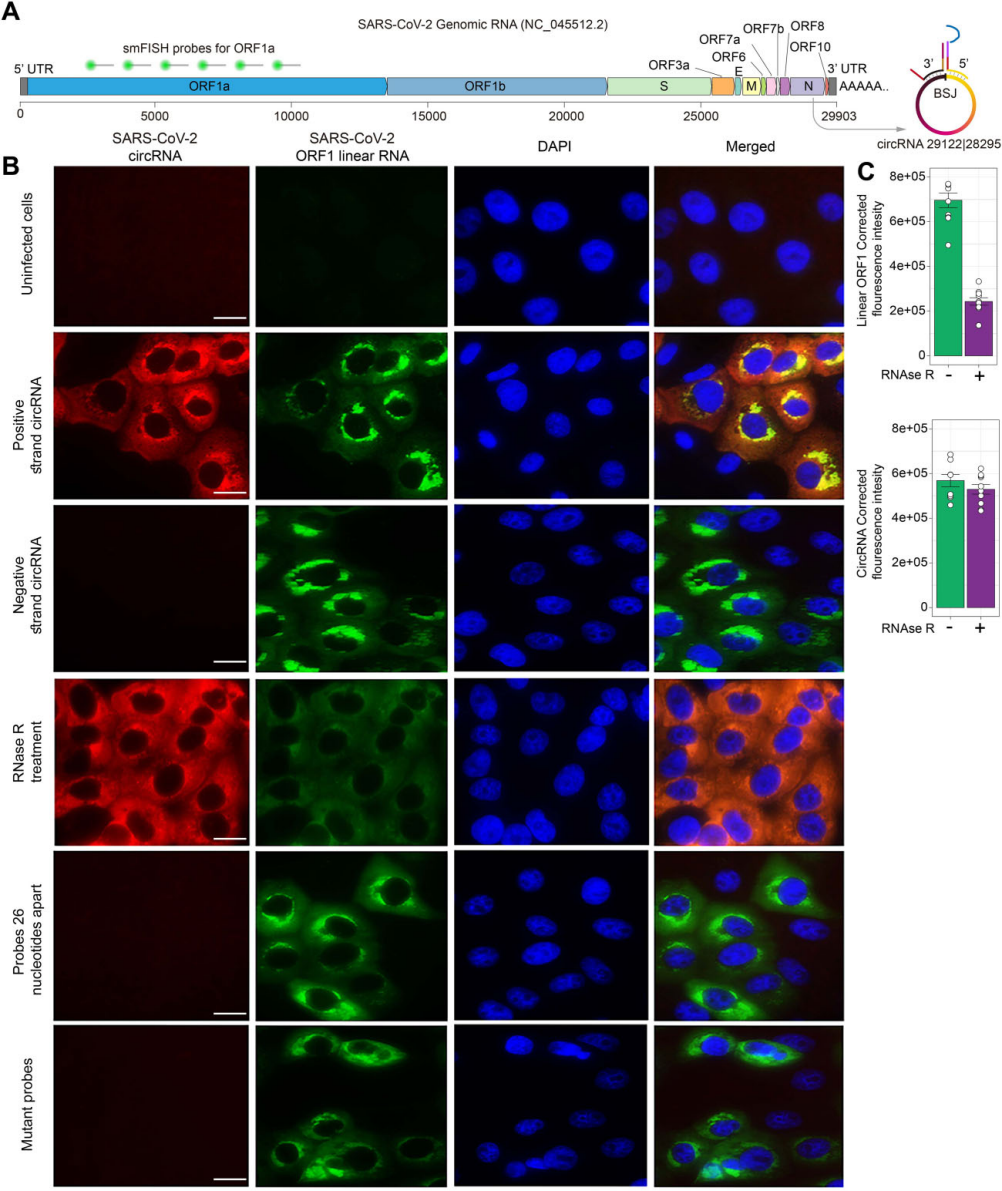
Imaging a circular RNA encoded by SARS-CoV-2 together with its genomic RNA. (**A**) Graphical representation of organization of SARS-CoV-2 genome, locations of the smFISH and ampFISH probes. circular RNA29122/28295 is created from the N-gene sequence during the replication process. (**B**) In the multiplex images the genomic RNA was imaged by hybridizing a set of 48 smFISH probes labeled with Texas Red (green) and the circular RNA was imaged by hybridizing an ampFISH probe pair followed by HCR using hairpins labeled with Cy5 (red). DAPI was used to stain the nuclei. The conditions and the probes used are indicated on the left and the targets are indicated on top. All infected cells display strong signals for genomic RNA but the bystander cells don’t show any. The target complementary region of each ampFISH probe in the bottom panel contains three mutations which will prevent their binding; and the target complementary regions of the probes in the second panel from the bottom bind 26 nt away from each other which will not allow them to interact. (**C**) Integrated fluorescence intensities within infected cells for genomic RNA and circular RNA with and without RNase R treatment. Data represent mean ± SE, *n* = 6 cells. A white line in each image represents a scale bar of 20 μm.

We designed a pair of donor and acceptor probes for each of the positive and the negative strand sequences at the BSJ. In parallel, we employed a set of smFISH probes for the SARS-CoV-2 genome Open Reading Frame 1a (ORF1a) (Figure 4A). These probes were hybridized to Vero cells (African green monkey cells) infected with SARS-CoV-2 (24 h post-infection). The ampFISH probes yielded a signal in Cy5 channel after HCR, whereas, the smFISH probes fluoresced in the Texas Red channel. The results show that infected cells show very strong signals for the positive strand circular RNA-29122|28295 and the SARS-CoV-2 genome (Figure 4). When the cells were treated with RNase R before hybridization, the signals from the genomic RNA largely disappeared, whereas the circular RNA signals remained the same (Figure 4B). The linear RNA of SARS-CoV-2 was so abundant that RNase R treatment could not fully eliminate the linear RNA from infected cells. However we noticed a significant (75%) decrease in the fluorescence intensity of the linear RNA (ORF1) after treatment with RNase R (Figure 4B). Consistent with the earlier PCR based studies (36), we found that circular RNA-29122|28 295 corresponding to the negative strand was not present in the cells. The specificity of the signals is also evident in the observation that only infected cells yield signals, whereas the ones not infected yield no detectable signals even when they are neighbors of the infected cells.

It is important to note that we did not observe spot-like signals in either smFISH or ampFISH but observed ‘unusually strong’ signals that were diffused through the cytoplasm with extra strength in the perinuclear regions. This likely occurs because the SARS-CoV-2 infected cells produce extremely high amounts of viral RNAs, so much so that 65% of total sequencing reads in the infected cells are derived from SARS-CoV-2 RNAs (45). As a result, spot counting was not feasible and we had to use average fluorescence in cells for quantification (Figure 4C).

Next, we sought to explore the specificity of the ampFISH method by designing a pair of ampFISH probes that will bind near the BSJ of SARS-CoV-2 circular RNA but will be separated from each other by 26 nucleotides. These probes can bind to the target region in circular RNA, but will not be able to undergo the conformational reorganization in acceptor probe that results in HCR. Accordingly, we observed no HCR signal with them (Figure 4B). Next, we created three random changes in the target binding regions of the acceptor and donor probes. As a result of these mutations, they would not be able to bind to the target sequence of the circular RNA. Accordingly, the mutated ampFISH probes yielded no signals (Figure 4B). These results verify the specificity of ampFISH in the imaging of N-gene circular RNA in virus-infected cells. Enlarged merged images of each panel in Figure 4 are presented in Supplementary Figure S4.

### Imaging a human cytomegalovirus (HCMV) derived circRNA in infected cells

Similar to SARS-CoV-2, we recently identified many circular RNAs in HCMV-infected cells through bioinformatic analysis of transcriptome of the virus-infected cells (46). Some of these circular RNAs were confirmed by sequencing of back-spliced junction regions (46). We focused on a well-characterized HCMV circular RNA, circular RNA number 5 (FJ616285.1:30 092|29 489) (code before the colon represents GenBank accession number of HCMV and after represents the location of BSJ on the HCMV genome). We designed a pair of ampFISH probes for the positive strand for the circular RNA detection and as a control, we designed a pair of ampFISH probes for the negative strand sequences, just as we did for the SARS-CoV-2 circular RNA. As a positive control, we also prepared a pair of ampFISH probes that bind to a linear HCMV transcript, UL24 (sequences provided in Table 1).

**Figure 5.**
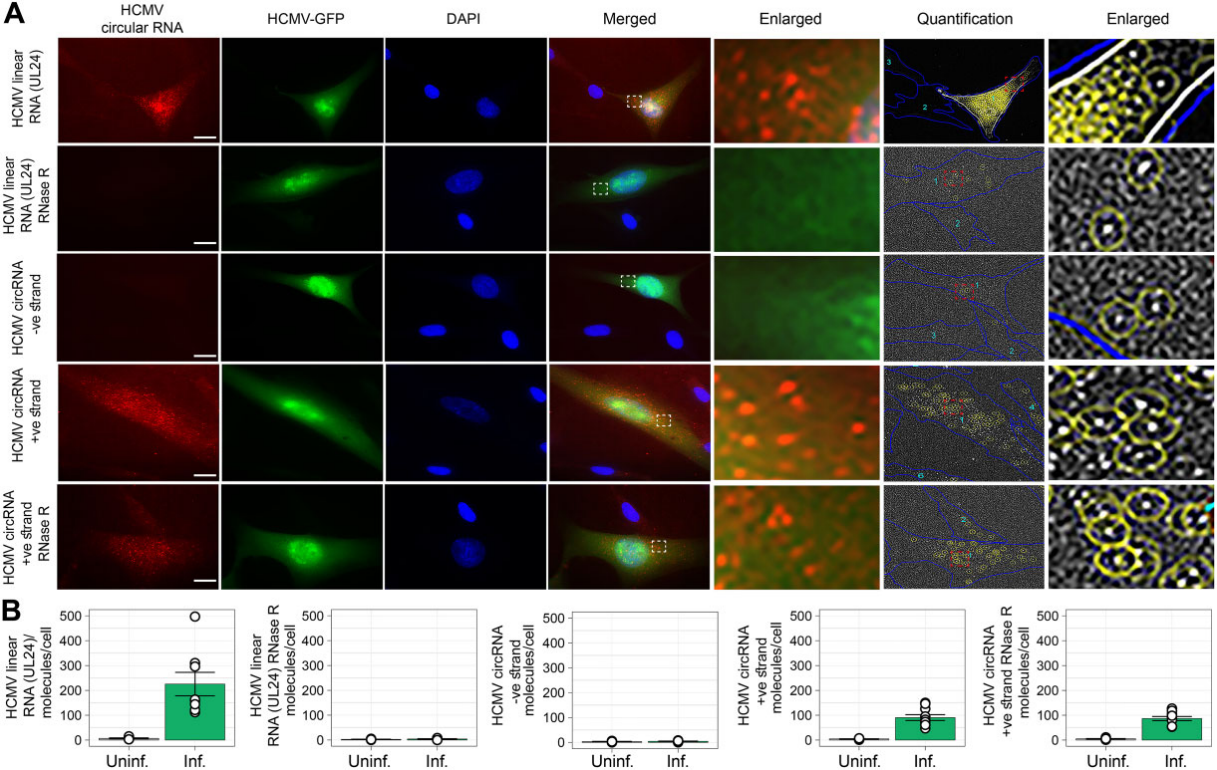
Imaging an HCMV circular RNA, circular RNA number 5, and an HCMV linear RNA transcript UL24, in HCMV-infected MRC-5 cells with ampFISH. (**A**) The probes employed are indicated in the left and the targets are indicated on the top. The smFISH RNA signals are represented by red color, HCMV-GFP infected cell by green, and DAPI stain by blue. Right two columns show the output of spot detection image processing, where yellow ovals are drawn around the detected spots and blue outlines are drawn around each cell. (**B**) The graphs show the average number of RNA molecules/cell ± SE, *n* = 10 cells. A scale bar of 20 μm is present in the images.

Our results show that ampFISH probes specific to the linear HCMV transcript UL24, as well as HCMV circular RNA5, produce spot-like signals in HCMV-infected cells but not in uninfected cells present in their neighborhood (Figure 5A). The HCMV-infected MRC-5 cells could be discerned by the expression of GFP since the viral construct that we used encodes GFP (47). RNase R treatment degraded the linear RNA and abrogated its signals but signals from the circular transcript remained unaffected (Figure 5A). Counts of spots in single infected, uninfected, RNase R treated and untreated cells confirmed the specificity of signals over many cells (Figure 5B). Enlarged merged images of Figure 5A are presented in Supplementary Figure S5.

### HCMV infection induced upregulation of circHIPK3(2) RNA

**Figure 6.**
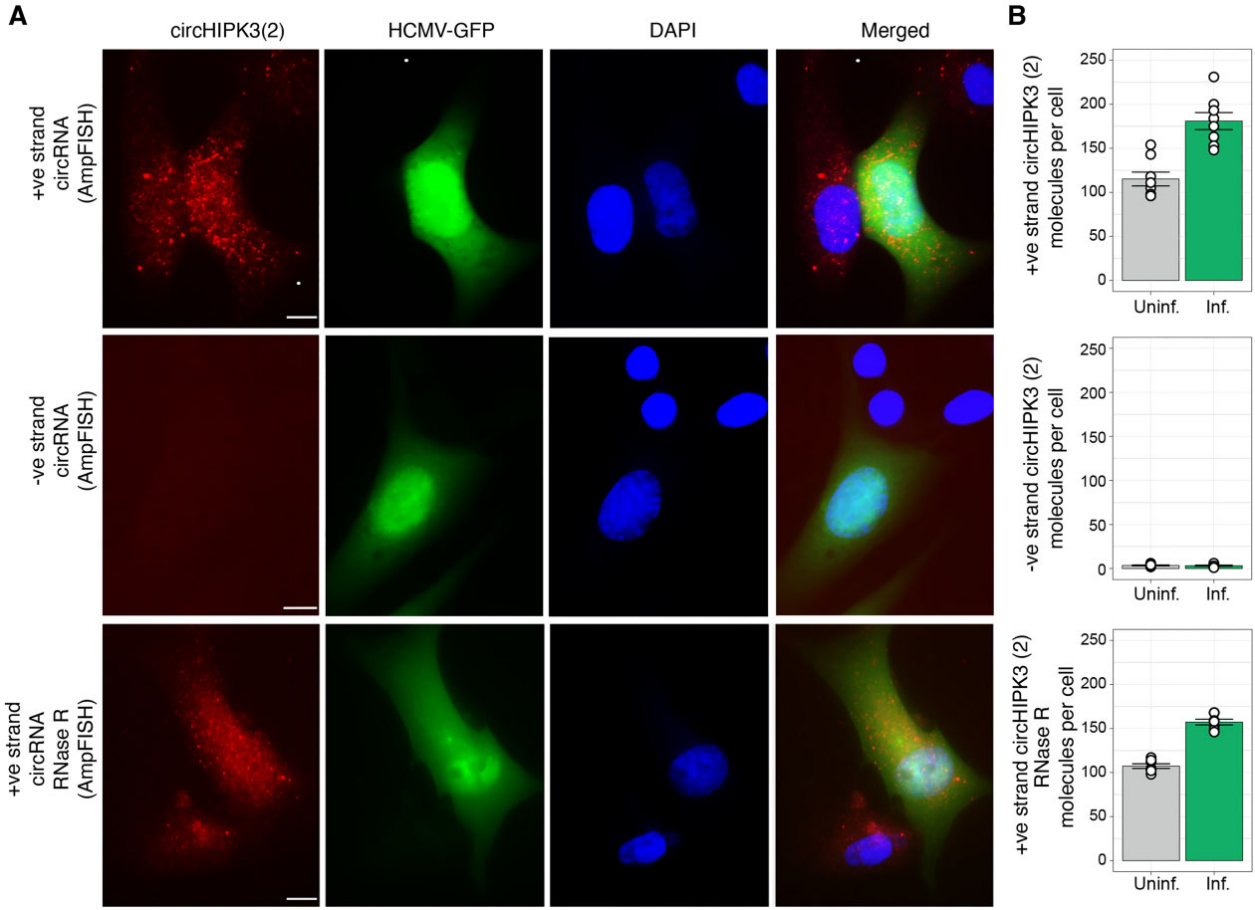
Upregulation of expression of human circular RNA, circHIPK3(2) in HCMV infected cells. (**A**) MRC-5 cells in a field where some cells are infected with HCMV and some are not, were imaged using ampFISH probes against human circular RNA. The ampFISH probes are indicated on the left and the labels are shown on the top. The HCMV infected cells can be identified by GFP expression (green). The ampFISH signal is shown in red. (**B**) The ampFISH spots were quantified in each kind of cells and are presented as bar graphs for the average molecules per cell for each indicated categories. Error bars are ± SE, *n* = 10 cells and size bars represent 10 μm.

Finally, we explored whether infection by HCMV has an impact on the levels of human circular RNA and whether imaging can quantify the changes in expression. Such studies are best done through imaging since single infected and uninfected cells that can be compared side by side. We imaged circHIPK3(2) RNA using the probes described above but performing the study on MRC-5 cells infected with an HCMV strain that expressed GFP. The infected cells showed increased expression of circHIPK3(2) than uninfected cells (Figure 6). These results are consistent with previous findings that show that expression of circHIPK3(2) circular RNA is increased during virus infection (26,48). Moreover, a negative strand of circHIPK3(2) was not detected in infected or uninfected cells (Figure 6). Furthermore, RNase R treatment could not eliminate the circHIPK3(2) in both infected and uninfected cells suggesting true circRNA molecules are detected by ampFISH (Figure 6).

## Discussion

Here, we demonstrate single molecule imaging of three different circular RNAs *in situ* using ampFISH while providing several lines of evidence for the specificity of their detection. The evidence included the following observations. (i) When the cells were treated with RNase R, which selectively degrades linear RNAs, the spots corresponding to the circular RNA molecules persisted, while those corresponding to their linear counterparts disappeared. (ii) Consistent with bioinformatic analysis of RNAseq data, probes specific to the positive strand of circular RNA sequence yielded signals and negative strand specific probes did not. (iii) In the case of two circular RNAs that were encoded by RNA viruses, only the virus infected cells yielded signals while uninfected cells did not. (iv) In the case of human RNA, circHIPK3(2), the number of spots in cells increased with HCMV infection, which is consistent with known upregulation by the infection process. (v) When the target binding regions of the probes were mutated or, moved by a few nucleotides, the ampFISH did not yield any signal. (vi) Digestion of all RNAs by RNase A treatment abrogated the signals. (vii) The circular RNA spots did not colocalize with the spots created by their linear counterparts.

The intensity of ampFISH spots for circular RNAs utilizing a single pair of probes was similar to the intensity of spots created by 48 smFISH probes and were accurately detected using our image processing programs (32). However, our previous work Marras *et al.* (32) indicates that when a single pair of ampFISH probes are employed, only a fraction (10–30%) of all the RNAs present in the cells are detected. On the other hand, when multiple pairs of ampFISH (or directly labeled smFISH probes) are tiled over the length of RNA, almost all RNAs (85%) can be detected (29,32). This gain by tiling of multiple probes over the RNA likely occurs because secondary structures or protein binding render some local sites impervious to probe binding but by using many probes binding to the same RNA it becomes possible to detect all RNAs. Since it is possible to use only one probe pair for circular RNA detection, we may have to contend with a fractional detectability. However, it may be possible to maximize the detectable fraction by using artificial nucleotides, like LNAs, that strengthen the probe binding so that the probes can compete more effectively with local secondary structures. Additionally, possibilities are to use ‘accessory’ probes that facilitate binding of ampFISH pairs by binding to part of the impervious secondary structure.

Among other technologies that are also suitable for detection of circular RNAs are two technologies that use a pair of probes binding at adjacent sites for signal generation. One of them is the ‘double Z’ probes in RNAscope technology (30) and the other is split HCR initiator in version 3 of HCR (49). In the former two oligonucleotide probes bind side-by-side on a target and then bind to two adjacent locations on a preamplifier probe which in turn bind to signal probes producing an amplified signal. This system of serially introduced probes is based on branched DNA amplification. In the third generation HCR, two oligonucleotide probes bind to the target at adjacent sites but the probes are oriented in a different manner. Their arrangement on the target looks like two U’s that touch each other while facing away from each other. This arrangement creates a two-part sequence upon binding to target that can initiate an HCR reaction. Detection of circular RNA through the RNAscope technology has been published (31), whereas, such circular RNA detection using the version 3 HCR is yet to appear. The requirement that the target sequences be immediately adjacent to each other is more strict in the case of version 3 HCR than it is in double z probes. Therefore, assays based on version 3 HCR may be as accurate as the one we describe using ampFISH where two probes must bind in the immediate vicinity of each other.

It is possible to implement ampFISH in multiple fluorescent colors for the detection of multiple circular RNAs or to develop controls where a linear RNA and circular RNA is detected at the same time, as we show in Figure 3. It is also possible to perform ampFISH together with smFISH as well as with immunofluorescence to include additional markers in imaging.

A reliable, high-resolution, single molecule FISH-based circular RNA detection whose design is publicly accessible will aid in the studies aimed at the mechanisms of circular RNA biogenesis as well as in the studies aimed at the functions of circular RNAs. Since in situ imaging with ampFISH can be performed in cells in culture, in tissue sections as well as in whole embryos of small animals, they will be useful in many biological contexts.

A limitation of our procedure is that the number of targets that can be detected is relatively few and is limited by the available orthologous HCR hairpins. Another limitation is that the newly designed probes for circRNA detection have to be validated with RNAse R treatment and the probe concentrations have to be optimized.

## Supplementary Material

gkae583_Supplemental_File

## Data Availability

All the data is available in the manuscript files. Figures 1–5 and 6 include the data. The probe sequence is provided in Table 1 and S1 Table.
