# Supplementary material for: Imaging and quantification of human and viral circular RNAs
Source: Nucleic Acids Res. 2024 Jul 25;52(15):e70. doi: 10.1093/nar/gkae583 (PMC11347131; doi:10.1093/nar/gkae583)
Supplement: gkae583_Supplemental_File [file gkae583_supplemental_file.docx]

**Supplementary figures**


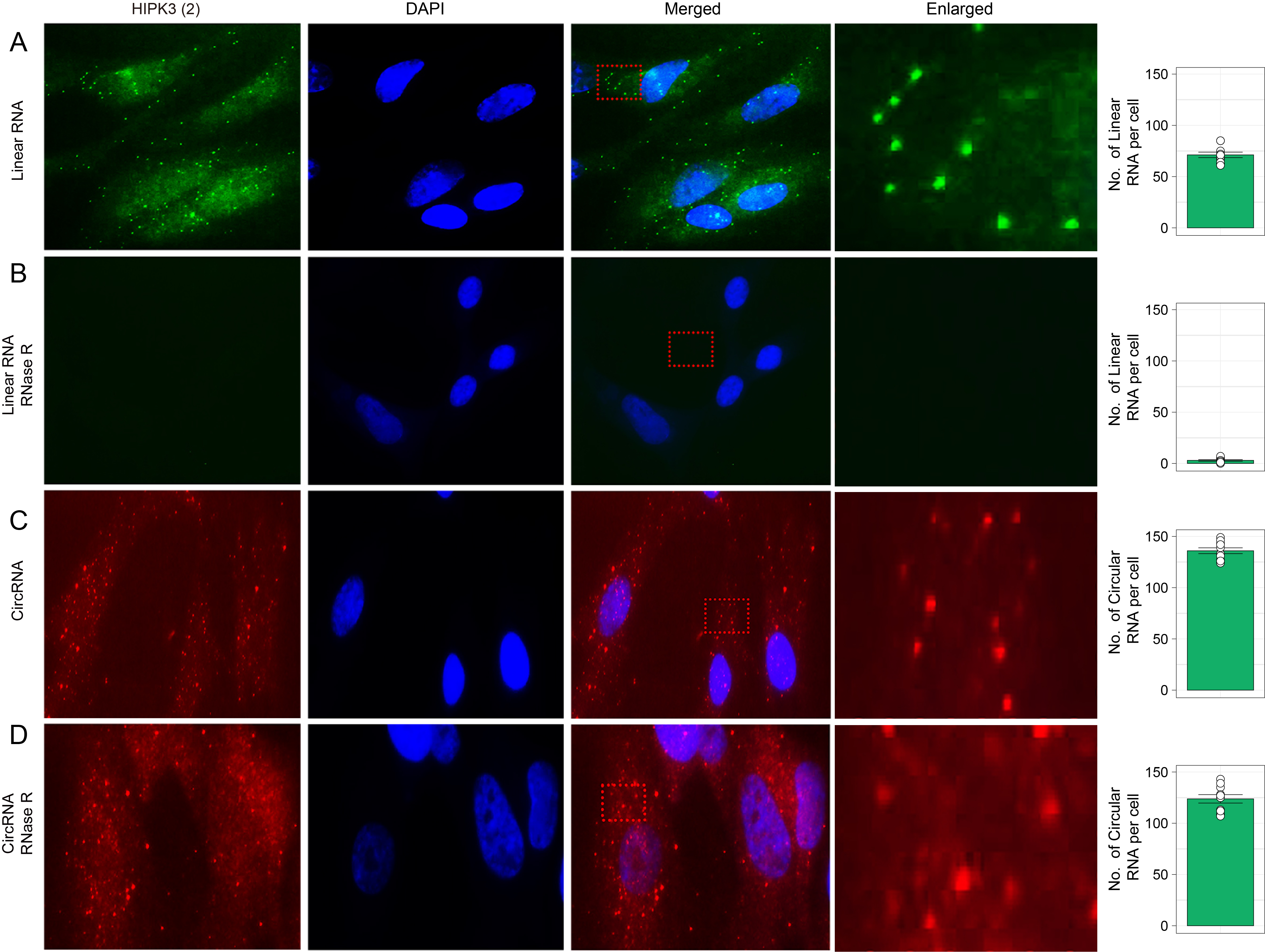


**Fig. S1. Imaging of HIPK3 linear RNA (green) and its circular RNA, circHIPK3(2) (red), in human lung fibroblast cells, MRC5**. A. smFISH imaging of HIPK3 linear RNA. B. Cells were treated with RNase R and stained with smFISH probes specific for HIPK3 linear RNA. C. circHIPK3(2) RNA imaged using ampFISH. D. Cells were treated with RNase R followed by imaging for circRNA via ampFISH. Merged represents an overlay of RNA and DAPI images. Each bright spot in the RNA images represents an RNA molecule. Enlarged represents area indicated by dotted square. Graphs on the right show number of spots in single cells as measured by our image processing program. Data is represented as mean ± SE. n= 10.

**
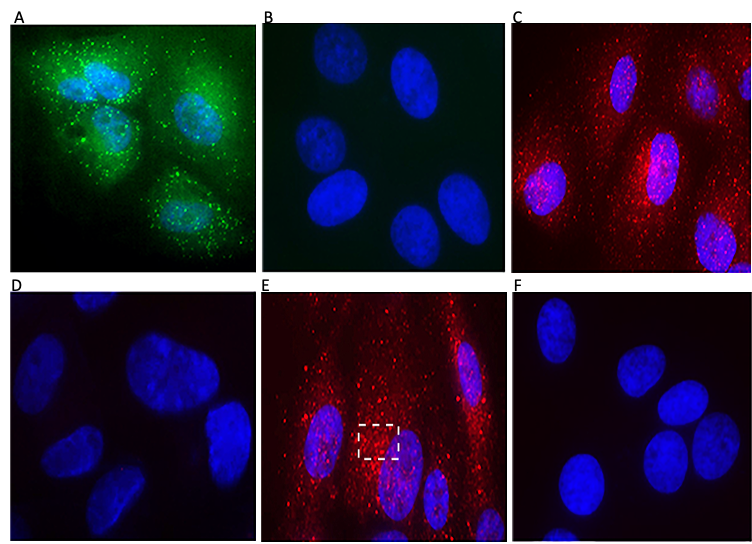
**

**Fig. S2.** **Enlarged versions of images of Figure 1C, column 3. A.** Enlarged version of image in Fig. 1C top, column 3. HIPK3 Linear RNA imaging using smFISH. Each green spot corresponds to a linear RNA of HIPK3. **B.** Enlarged version of the second image from the top (RNase R treatment). RNase R treatment removes the linear RNA of HIPK3. **C.** Enlarged version of the third image from top. Imaging of circRNA of HIPK3 in ARPE-19 cells using ampFISH. Each red spot corresponds to circRNA of HIPK3. **D.** Enlarged version of the fourth image from top. Negative strand circRNA of HIPK3 was not detected. **E.** Enlarged version of the fifth image from top. CircRNA of HIPK3 was resistant to RNase R treatment. **F.** Enlarged version of the sixth image from top. RNase A treated cells did not show circRNA of HIPK3.


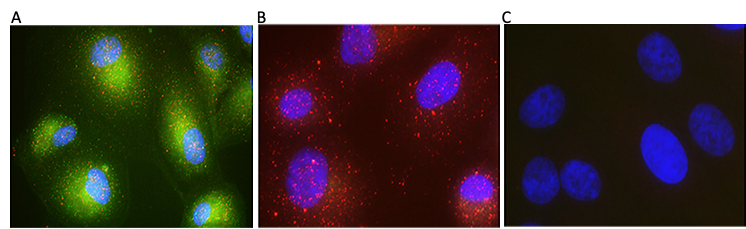


**Fig. S3. Enlarged versions of images in Figure 2 column 4**. **A.** Enlarged version of the image in top panel, column 4. Co-imaging of circRNA and linear RNA of HIPK3 in ARPE-19 cells. Red spots correspond to circular RNA and green spots represent linear RNA of HIPK3. **B.** Enlarged version of the image in middle panel, column 4. RNase R treatment removes the linear RNA but not circRNA. Each red spot corresponds to a circHIPK3(2). **C.** Enlarged version of the image in bottom panel, column 4. RNase A treatment removes the circRNA and linear RNA of HIPK3. More details are described in the figure legend of Fig. 2.


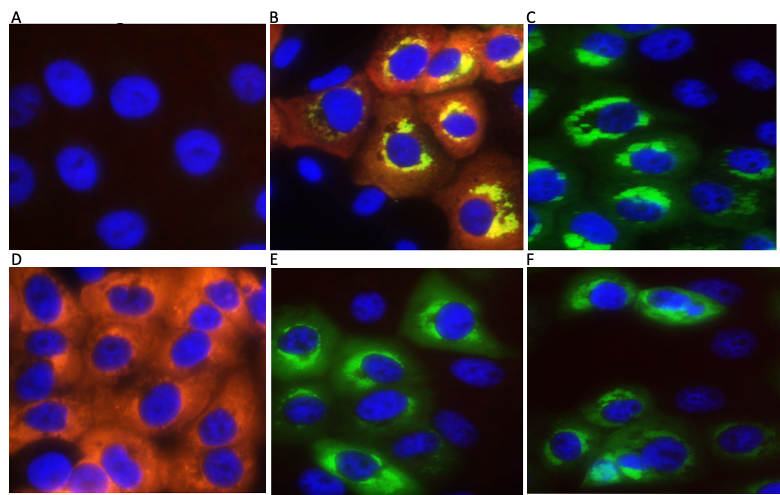


**Fig S4. Enlarged versions of images in Figure 4B, column 4.** **A.** Enlarged version of the image in top panel, column 4 in Figure 4B. Uninfected Vero cells did not show SARS-CoV-2 RNA. **B.** Enlarged version of the image in second from the top panel, column 4. Infected Vero cells show the virus-specific circRNA and linear RNA. **C.** Enlarged version of the image in third from the top panel, column 4. A negative strand of N-gene circRNA was not detected in the infected cells. **D.** Enlarged version of the image in the fourth panel from the top, column 4. RNase R treatment of cells specifically reduces the linear RNA level. **E.** Enlarged merged of the fifth image from top. N-gene circRNA was not detected when the acceptor and donor probes were 26 nucleotides apart at the BSJ region. **F.** Enlarged version of the image in the bottom panel, column 4. N-gene circRNA was not detected by ampFISH when three random mutations were introduced in ampFISH probes. The green signal corresponds to the linear RNA of ORF1a (smFISH) and the red signal represents the circRNA of N-gene (ampFISH).


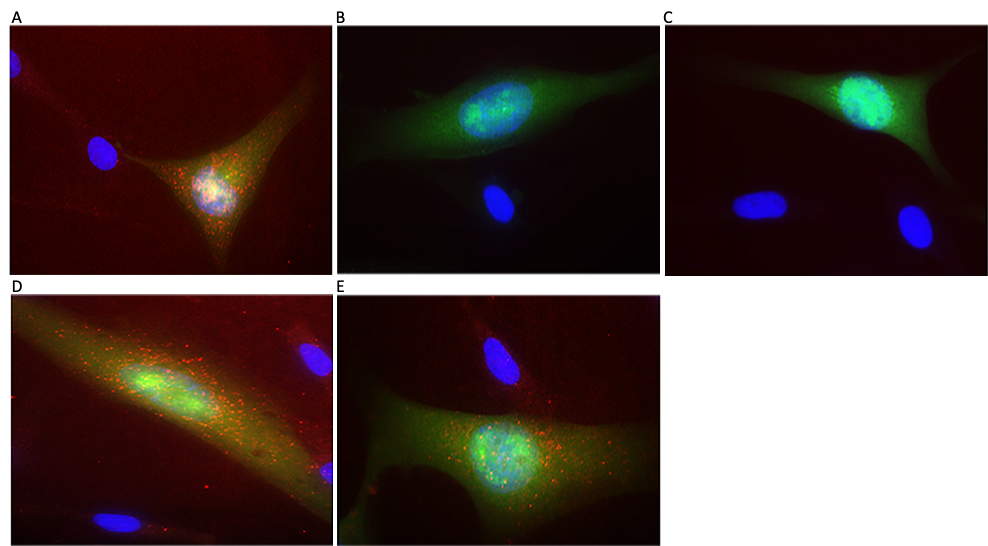


**Fig. S5.** **A.** **Enlarged versions of the merged images of Figure 5A, column 4**. **A.** Enlarged version of the image in the top panel, column 4 in Figure 5A. HCMV linear RNA was is detected by ampFISH in only virus-infected cells. **B.** Enlarged version of the image in second from the top panel, column 4 in Figure 5A. RNase R treatment removes the HCMV linear RNA. **C.** Enlarged version of the image in third from the top panel, column 4 in Figure 5A. Negative strand of HCMV circRNA was not detected. **D.** Enlarged version of the image in fourth from the top panel, column 4 in Figure 5A.. HCMV circRNA imaging using ampFISH. **E.** Enlarged version of the image in the bottom panel, column 4 in Figure 5A. HCMV circRNA after RNase R treatment. Green signals represent the HCMV-infected cells. The red signal represents the circRNA of CMV and the green GFP expression.

**Table S1:** Sequences of single molecule FISH (smFISH) probes for HIPK3 and SARS-CoV-2 ORF1a.

| S.N. | HIPK3 smFISH probe sequences | SARS-CoV-2 ORF1a smFISH probe sequences |
| --- | --- | --- |
| 1 | caaaaggcacttgactgagttt | tgagttggacgtgtgttttc |
| 2 | tggtatctttgtctgaaaagca | gcgaacctgtaaaacaggca |
| 3 | cagcacttgtctgcaatgaaaa | ccataacatgaccatgaggt |
| 4 | acaattcatcgacaatctgcat | tactgaatgccttcgagttc |
| 5 | cttcatgctgtactaactgata | aatgcactcaagagggtagc |
| 6 | ccagcatctcaaagactaaaca | ccagttgttcggacaaagtg |
| 7 | aggatccaccaacataatattc | tttcagaacgttccgtgtac |
| 8 | ctattactttaaccctgtaagg | tggtgacgcaactggataga |
| 9 | gtagtaccgagattgtagatat | gaaaggcacatttggttgca |
| 10 | ccatccaagaaataattctgca | tggctttaacaaaatcgccc |
| 11 | gatttagtacccacatttaaca | agtaaccacaagtagtggca |
| 12 | agacatatctgtttctttgcaa | tccaaaggcaatagtgcgac |
| 13 | aaagatcacttccttccaaatc | cctgtatggttacaacctat |
| 14 | caaattctcttctatcagcttt | cattaagaccttcggaacct |
| 15 | aatctgcatcaatcagcaacat | actgaacaacaccacctgta |
| 16 | gtgggacttacaaatatccata | tcaaggacgggtttgagttt |
| 17 | aactgtattatctacccttatt | accttccttaaacttctctt |
| 18 | aatgctgaatgtgggatattgg | gaatgtctgaacactctcct |
| 19 | cacaatctagactacctttaca | acttctgtgggaagtgtttc |
| 20 | tcttcatctgacatactattcg | acagcttcactagtaggttg |
| 21 | cacaaaagtgctctctgcaaat | acaaactggtgtaccaacca |
| 22 | ataccaattctgtgttttcatg | atcatattaggtgcaagggc |
| 23 | gatgggtatggaagtagagtag | aagtaacctttgttggtgca |
| 24 | taacataggtcttgaggtacac | gtcttgttgaccaacagttt |
| 25 | atggtaacagacggggatttaa | cttatttaaggctcctgcaa |
| 26 | ttttgttggactcagtggaaat | cacagcagttaaaacaccct |
| 27 | tttcacatatatggatactggc | ttggcacttttctcaaagct |
| 28 | aagttgtttaaattgcacccaa | tacatagccaagtggcattg |
| 29 | acttgtatgctgtaattctgac | ctagttgtgtagattgtcca |
| 30 | agacagcagaattcagctatag | acacttttatcacctctctt |
| 31 | taactgcttgcttttcttgaag | tctaggtggaatgtggtagg |
| 32 | gtttgcccaagctaagataaaa | atgtcattgacatgtccaca |
| 33 | tgcacacaaagaactcacatct | gctccatccaaataagttgg |
| 34 | agcaaacatcaccaacaagcaa | gtggtagtactcaaaagcct |
| 35 | tttggtgctgtaaaagcaactt | gggtatttccactttttagt |
| 36 | tctatgcaaaaagtagctacct | cttgtagagcaggtggatta |
| 37 | ccacctttacactacaatatac | ccacaagttttacacaccac |
| 38 | gctaaaacacagcctcaattta | accacactggtaattaccag |
| 39 | cgttatatagcagcttatctat | gtaaagcaccgtctatgcaa |
| 40 | ttaatcacctaatttcacagct | cttgcgtttggatatggttg |
| 41 | caaatgcaattattttccacca | gtatacaccaggtatttggt |
| 42 | atctagagttttacactccaat | ctggttttagatcttcgcag |
| 43 | atccattattagcttattgtga | aagcagcggttgagtagatt |
| 44 | tgcttgtgttaatggagtgaag | acaaccgtctacaacatgca |
| 45 | acttgtggggaaaattaagtcc | tacattcgactcttgttgct |
| 46 | tttgtacctgttgtgatacaat | ctttacctccattagcatag |
| 47 | agtccttattgtaatattgcac | gtactaccagcacagaatgt |
| 48 | ctatcgcaaaccgtaatagaca | agtgacaagtctctcgcaac |
